# Stage-Specific Differences in Fungal Community Structure and Functional Potential During Litter Decomposition in a Lava Plateau

**DOI:** 10.3390/microorganisms14071581

**Published:** 2026-07-20

**Authors:** Yan Zhu, Jiaxing Huang, Yingjun Ye, Zhichao Tian, Jianhui Jia, Yueyu Sui, Yanli Zhang

**Affiliations:** 1 School of Life Science and Technology, Mudanjiang Normal University, Mudanjiang 157011, China; 2 College of Hydraulic Science and Engineering, Northeast Agricultural University, Harbin 150030, China; 3 State Key Laboratory of Black Soils Conservation and Utilization, Northeast Institute of Geography and Agroecology, Chinese Academy of Sciences, Harbin 150081, China

**Keywords:** lava plateau, stand type, litter, decomposition stage, fungi, community structure, co-occurrence network, LEfSe analysis, functional prediction

## Abstract

Litter-inhabiting fungi drive organic matter mineralization, regulate nutrient cycling, and support ecosystem stability. Understanding their dynamics in unique geological habitats is essential for predicting ecological recovery on volcanic landforms. Using high-throughput ITS sequencing and physicochemical analyses, we investigated litter-inhabiting fungal communities across four stand types on the Jingpo Lake lava plateau—shrub forest (SF), deciduous broad-leaved forest (DB), coniferous and broad-leaved mixed forest (CB), and coniferous forest (CF)—at the early (t_1_) and late (t_2_) stages of decomposition. The results showed significant differences in litter physical and chemical properties among forest stand types (*p* < 0.05). Regarding community composition, Ascomycota and Basidiomycota dominated throughout, and the core genera were primarily *unclassified_o__Helotiales*, *Mortierella*, and *unclassified_k__Fungi*. Alpha diversity analysis showed that DB had the highest Shannon and Pielou-e indices at stage t_1_, while CB exhibited higher OTUs and Chao1 indices at stage t_2_. Beta diversity showed that SF communities were significantly separated between the two stages. Co-occurrence networks showed the highest connectivity in CF with pronounced modularity. Notably, LEfSe analysis revealed that DB had the fewest biomarkers, suggesting matrix heterogeneity suppresses single-taxon dominance. Functionally, saprotrophs dominated initially but transitioned toward complex soil saprotroph and endophyte assemblages over time. Redundancy analysis (RDA) identified litter moisture content (LMC) and carbon (C) content as primary drivers, orchestrating a systematic shift in community assembly from “moisture-driven colonization” at t_1_ to “carbon quality screening” at t_2_. These findings provide a microecological basis for understanding plant-litter-microorganism coupling mechanisms and guiding ecological restoration in lava plateau ecosystems.

## 1. Introduction

Litter decomposition is a central process controlling turnover of the surface organic carbon pool in forest ecosystems and directly drives transfer of nutrients from plant residues into the soil pool [1]. This biogeochemical cycle not only sets the mineralization rate of organic matter but also controls the release patterns and recycling efficiency of key elements such as nitrogen and phosphorus [2]. This process is shaped jointly by stand type, litter substrate quality, and microorganisms, and it displays clear stage-specific characteristics [3]. These successive stages are fundamentally driven by predictable physicochemical trajectories: the early stage (t_1_) is characterized by rapid leaching of water-soluble compounds, high moisture availability, and the swift depletion of labile carbon and nutrients, which favors opportunistic microbial colonizers; conversely, the late stage (t_2_) is marked by the relative enrichment of recalcitrant structural polymers (e.g., lignin and cellulose), accumulation of secondary metabolites, and intensified nutrient limitation, exerting strong environmental filtering on specialized decomposers [4,5]. Understanding the microbial drivers of this process is crucial for assessing forest ecosystems’ carbon sequestration and nutrient retention capacities.

Microbial communities, as the agents of decomposition, drive the breakdown of organic macromolecules and elemental cycling by secreting extracellular enzymes [6]. Fungi, compared with bacteria, play an irreplaceable and often dominant role in litter decomposition because their extensive mycelial networks and specialized metabolism enable efficient degradation of recalcitrant structural components such as lignin and cellulose [7]. As the primary inhabitants of the litter microhabitat, these litter-inhabiting fungi dynamically respond to substrate degradation. In the early stage of decomposition, fungal communities are driven mainly by readily available carbon sources and are dominated by broad-spectrum saprophytic fungi. As labile compounds are depleted and recalcitrant components such as lignin and cellulose become relatively enriched, the community structure is substantially reorganized, and lignin-degrading fungi and substrate-specific decomposers gradually become dominant [5]. This successional process involves not only shifts in species composition but also changes in the relative abundances of fungal trophic functional groups (for example, saprotrophs, ectomycorrhizal fungi, and pathogens), alterations in the topology of hyphal interaction networks, and modifications to the potential for carbon and nitrogen metabolism [8]. Therefore, a comprehensive investigation of fungal community composition, nutritional succession, and functional potential is essential to elucidate the microbial mechanisms underlying litter decomposition.

The Jingpo Lake lava plateau has a distinctive volcanic geology, and its soils are young, shallow, and nutrient-poor, creating a unique microbial habitat [9]. Globally, studies across various volcanic ecosystems have consistently demonstrated that litter decomposition in such nutrient-limited environments is a highly phased process. For instance, research on Hawaiian chronosequences and Mount Etna has shown that fungal communities undergo pronounced successional shifts, transitioning from early-stage colonization by fast-growing taxa utilizing labile carbon to late-stage dominance by specialized decomposers targeting recalcitrant compounds [10,11]. Similarly, studies on the unique lava-formed Gotjawal forests in Jeju Island have revealed that volcanic substrates impose strong environmental filtering on microbial community assembly and functional potential, particularly in nutrient cycling [12]. These findings provide a clear theoretical rationale that microbial assembly during decomposition is fundamentally constrained by the dynamic physicochemical evolution of volcanic substrates. Although prior work has characterized soil fungal communities in this region, revealing that Ascomycota and Basidiomycota are the dominant taxa and that a core microbiota (comprising specific saprotrophic and symbiotic genera) persists across different stand types despite stand-specific variations [13], systematic evidence is still lacking on how litter-inhabiting fungal communities dynamically evolve during the decomposition process on lava plateaus. Especially in the early and late stages of litter decomposition, it remains unclear whether the fungal community shifts from a “phyllosphere-associated” to a “soil-associated,” and how its metabolic functional potential responds to the nutrient-limited environment of volcanic soils [14,15,16]. Although recent studies have documented rapid fungal succession during litter decomposition [15] and the transition from phyllosphere to soil-derived communities [16], and have explored microbial responses in volcanic ecosystems [17], systematic evidence regarding the stage-specific shifts in fungal community assembly and functional potential during litter decomposition on lava plateaus remains limited [16]. These gaps leave critical uncertainties in current research on microbial ecology in volcanic ecosystems. This study focuses on the Jingpo Lake lava plateau and compares fungal community structure between the early and late stages of litter decomposition. It characterizes the successional patterns and functional potential of fungal communities on this unique substrate and identifies the key physicochemical factors driving community shifts. Specifically, we aimed to test the following hypotheses: (1) The litter-inhabiting fungal community undergoes a deterministic successional shift from being dominated by “phyllosphere-associated” taxa (fast-growing saprotrophic Ascomycota driven by moisture availability) in the early stage to being enriched with “soil-associated” taxa (lignin-degrading Basidiomycota and symbiotic/endophytic guilds driven by recalcitrant carbon quality) in the late stage. (2) Stand-specific litter substrate quality significantly dictates fungal network topology and functional potential; specifically, mixed litter substrates suppress single-taxon dominance through high substrate heterogeneity, whereas coniferous substrates select for fungal communities with higher network complexity and more conservative metabolic strategies. Ultimately, this research aims to elucidate the microbial mechanisms driving litter decomposition in volcanic ecosystems and to provide a theoretical foundation for vegetation restoration and nutrient management on lava plateaus.

## 2. Materials and Methods

### 2.1. Site Profile

**Figure 1:**
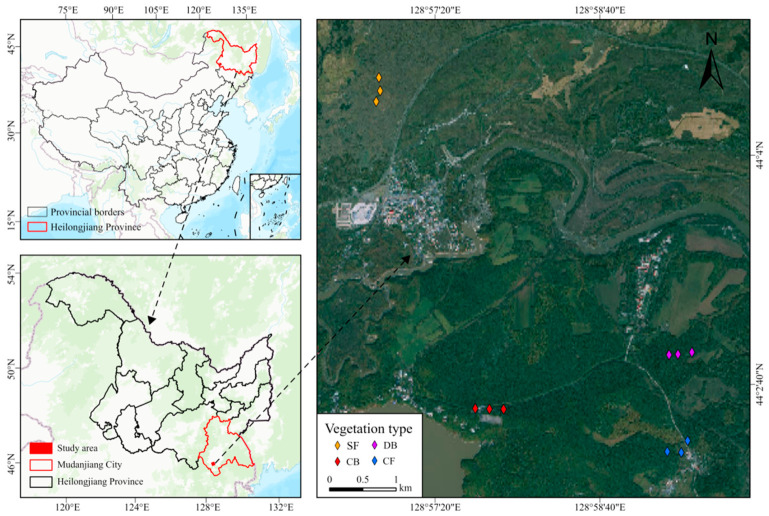
Study area of Jingpo Lake World Geopark in Heilongjiang Province, China.

The study area lies on the Jingpo Lake lava plateau in Ning’an City, Heilongjiang Province (128°30′–129°10′ E, 43°46′–44°18′ N) and forms the core protected zone of the World Geopark (Figure 1). The area has a temperate continental monsoon climate, with an average annual temperature of 3.6 °C and mean annual precipitation of 506.4 mm [9]. Precipitation is concentrated in summer. The terrain consists of low mountains and hills, with elevations ranging from 339.17 to 1260.7 m. The basalt plateau covers approximately 200 km^2^. Its geological structure formed from volcanic eruptions between 5200 and 5500 years ago, and the dominant lithology is alkaline olivine basalt [18]. Weathering of the parent material produced volcanic stony soil and dark brown volcanic ash soil. These soils have coarse, stony textures and exhibit pronounced spatial heterogeneity in nutrient distribution.

### 2.2. Litter Collection and Treatment

The litter decomposition experiment was conducted in situ using the nylon mesh bag method. In September 2024, fresh litter of the dominant tree species was collected from four stand types in the study area: shrub forest (SF), deciduous broad-leaved forest (DB), coniferous and broad-leaved mixed forest (CB), and coniferous forest (CF) (Table 1). After air-drying to constant weight indoors, the litter was homogenized. Ten grams of the sample were placed in a nylon mesh bag (15 cm × 15 cm) with an upper mesh of 1 mm and a lower mesh of 0.5 mm. The mesh bag was sterilized by high temperature. For each stand type, three independent plots were established as replicates. In each plot, existing litter and debris were removed from the ground surface. Mesh bags were laid flat on the ground with at least 5 cm spacing between them and secured with plastic ground nails, while preserving the natural state as much as possible. During the experiment, we monitored mass loss continuously and operationally defined decomposition stages by mass-loss rate: the early stage (t_1_, ~30% mass loss) was selected to capture the phase of rapid labile carbon depletion and high moisture availability, while the late stage (t_2_, ~90% mass loss) represents the phase of recalcitrant compound accumulation and advanced substrate stabilization [14,19]. It is important to note that due to the distinct initial substrate qualities and microclimatic conditions across the four stand types, the actual time required to reach these specific mass-loss thresholds varied. However, sampling at equivalent mass-loss stages, rather than at fixed calendar intervals, is a widely accepted ecological approach. This strategy ensures that we are comparing functionally equivalent decomposition phases (i.e., similar biogeochemical states) across different stand types, thereby capturing the true substrate-driven successional dynamics rather than arbitrary temporal snapshots [20]. We used a non-synchronous sampling strategy. When the litter of a given stand type reached the preset threshold, we retrieved it immediately. We manually removed roots and soil particles from the collected material, homogenized the samples, and divided each into three portions. One portion was dried at 80 °C to constant weight to calculate decomposition rate. A second portion was air-dried and ground for analysis of physical and chemical properties. The third portion was placed in a sterile centrifuge tube, flash-frozen in liquid nitrogen, and stored at −80 °C for subsequent microbial sequencing of the litter. In total, 24 independent nylon mesh bags (4 stand types × 2 decomposition stages × 3 replicates) were deployed in the experiment. Consequently, the total number of independent biological samples used for calculating decomposition rates, conducting physicochemical analyses, and performing DNA extraction was 24 for each respective analytical category (i.e., *n* = 12 per decomposition stage).

### 2.3. Determination of the Physical and Chemical Properties of Litter

Litter moisture content (LMC) was determined by the drying method [21]. Litter pH was measured with a pH meter [22], and litter electrical conductivity (EC) was measured with a conductivity meter [23]. Litter carbon (C) content was determined by the potassium dichromate–external heating method [24], and nitrogen (N) content was measured by the Kjeldahl digestion method [25]. Phosphorus (P) content was determined by the molybdenum–antimony anti-colorimetric method [26]. Litter lignin (Li), cellulose (Ce), and hemicellulose (He) contents were measured using the Van Soest detergent fiber analysis method [27].

### 2.4. DNA Extraction, PCR Amplification, and Amplicon Sequencing

Microbial DNA from litter was extracted with a commercial DNA extraction kit (Shanghai Majorbio Bio-pharm Technology Co., Ltd., Shanghai, China). DNA integrity was evaluated by 1% agarose gel electrophoresis, and DNA concentration was measured with a UV spectrophotometer. The fungal ITS1 region was amplified using primers ITS1F (5′-CTTGGTCATTTAGAGGAAGTAA-3′) and ITS2R (5′-GCTGCGTTCTTCATCGATGC-3′). The PCR amplification was performed using a standard thermal cycling profile: initial denaturation at 95 °C for 3 min, followed by 35 cycles of denaturation at 95 °C for 30 s, annealing at 55 °C for 30 s, and extension at 72 °C for 45 s, with a final extension at 72 °C for 10 min. PCR products were visualized on an agarose gel and purified with AMPure XP beads (Beckman Coulter Genomics, Danvers, MA, USA) to remove primers and nonspecific fragments. The purified amplicons were then quantified and pooled. Sequencing was carried out on an Illumina MiSeq PE300 platform (Shanghai Majorbio Bio-pharm Technology Co., Ltd., Shanghai, China), yielding paired-end reads.

We applied quality filtering to ensure adequate sequencing depth. Sequences were retained only if they satisfied all of the following: (i) Phred quality score ≥ 30 (Q30, corresponding to a base-calling error rate ≤ 0.1%); (ii) merged read length between 200 and 400 bp; (iii) absence of ambiguous bases (N); and (iv) no chimeric sequences as detected by the built-in chimera removal algorithm. Negative controls were included in both DNA extraction and sequencing to monitor potential contamination.

Fungal sequencing data were processed in QIIME (version 2023.2). DADA2 performed quality trimming, denoising, sequence merging, chimera removal, and ASV (Amplicon Sequence Variant) inference. Taxonomic assignment used the RDP (version 11.5) Classifier against the UNITE (version 8.0) database, and species abundance per sample was calculated from the ASV abundance table. The annotation confidence threshold was set to 0.7.

We predicted and classified the ecological functional traits of litter-inhabiting fungal communities using the FUNGuild database on the Majorbio Cloud Platform. This method provided high-resolution functional profiles of fungal guilds in the absence of metagenomic sequencing.

### 2.5. Statistical Analysis of Physicochemical Properties and Alpha Diversity

Statistical analyses were performed using IBM SPSS Statistics 27.0, and graphs were generated using Origin 2021 (https://www.originlab.com/, accessed on 1 May 2026). Data normality and homogeneity of variance were evaluated using the Shapiro–Wilk and Levene’s tests, respectively. Data that did not meet these assumptions were log(x + 1) transformed prior to parametric analyses. Differences in litter physicochemical properties (LMC, pH, EC, C, N, P, Li, Ce, He) and fungal alpha-diversity metrics (Shannon, Simpson, Chao1, Pielou-e, Coverage) among stand types were analyzed using one-way analysis of variance (ANOVA), followed by Tukey’s honestly significant difference (HSD) post hoc test (α = 0.05). Additionally, the distributions of categorical variables were examined using Chi-square tests. The significance threshold for all tests was set at *p* < 0.05.

### 2.6. Bioinformatic and Multivariate Analyses of Microbial Community Structure

To ensure cross-sample comparability for microbial community evaluations, the raw ASV table was subsampled (rarefied) to an even depth of 77,056 reads per sample prior to analysis. This specific cutoff corresponded to the lowest valid sequence count post-quality control, confirming that every sample preserved adequate sequencing depth for reliable diversity profiling (with a Coverage index exceeding 97% across all samples).

To investigate the evolutionary relationships among fungal taxa at the genus level, a phylogenetic tree was constructed using the maximum likelihood (ML) method based on the representative sequences of the top 100 most abundant genera. For taxonomic annotation and visualization, standardized nomenclature was applied to unclassified taxa: each unclassified taxon was labeled with its highest resolved taxonomic rank prefix (k__, p__, c__, o__, f__, g__) followed by the last classified taxon name, ensuring consistent and interpretable labeling throughout the phylogenetic analysis. To infer fungal interrelationships and compare network topological variations across stand types, co-occurrence networks were generated based on the 100 most abundant ASVs across the entire sample set. This threshold was deliberately chosen to focus on the ecologically dominant taxa that drive major decomposition functions, while effectively minimizing spurious correlations and computational noise often introduced by rare, low-abundance taxa [28,29]. Networks were then constructed using Spearman’s rank correlation coefficients, retaining only strong associations (|r| > 0.6 and FDR-adjusted *p* < 0.05) through the Majorbio Cloud Platform, and visualized using Gephi 0.10.1 (https://gephi.org/, accessed on 11 May 2026). To identify taxonomic biomarkers and core communities that significantly differ among stand types, the linear discriminant analysis effect size (LEfSe) approach was utilized via the Majorbio Cloud Platform, applying a Kruskal–Wallis rank sum test threshold of 0.05 and a logarithmic LDA score cutoff of 2.0.

To quantify the associations between litter environmental variables and fungal community/diversity indices, Pearson correlation analysis was performed, corrected for multiple testing via the FDR method (significance threshold: *p* < 0.05). To illustrate the responses of fungal assemblages to environmental gradients, redundancy analysis (RDA) was executed in CANOCO 5 (https://www.canoco5.com, accessed on 16 May 2026). A preliminary detrended correspondence analysis (DCA) verified the suitability of a linear response model (gradient length < 3.0 SD) prior to modeling. Environmental predictors were standardized beforehand, and multicollinearity was evaluated using Variance Inflation Factors (VIFs); variables yielding a VIF > 10 were omitted to guarantee model robustness. Key explanatory variables were extracted through forward selection coupled with 999 permutation tests (FDR-corrected, *p* < 0.05).

Finally, to provide high-resolution functional profiles, the ecological functional traits of litter-inhabiting fungal communities across different stand types were predicted and classified using the FUNGuild database on the Majorbio Cloud Platform.

## 3. Results

### 3.1. Physicochemical Properties of Litter in Different Stand Types

**Figure 2:**
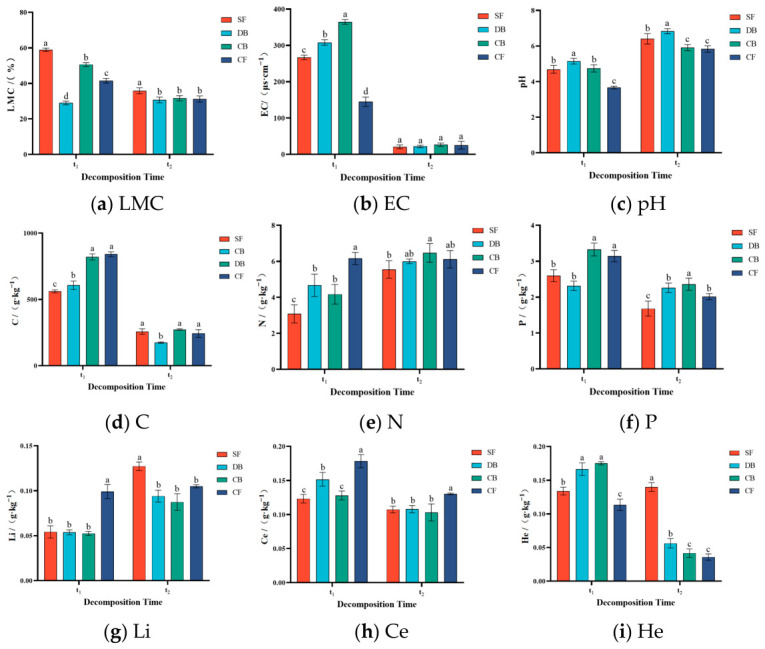
Physicochemical property characteristics of litter in different stand types. Note: LMC (litter moisture content), EC (electrical conductivity), C (carbon), N (nitrogen), P (phosphorus), Li (lignin), Ce (cellulose), and He (hemicellulose). SF (shrub forest), DB (deciduous broad-leaved forest), CB (coniferous and broad-leaved mixed forest), CF (coniferous forest). Different lowercase letters (e.g., a, b, c, d) above the bars indicate significant differences among the four stand types at the same decomposition stage (one-way ANOVA followed by Tukey’s HSD test, *p* < 0.05). Error bars represent standard deviation (SD); *n* = 3 per stand type.

Litter physical and chemical properties differed significantly among stand types at stages t_1_ and t_2_ (One-way ANOVA, *p* < 0.05; Figure 2). For water content, the LMC of SF was highest at both t_1_ and t_2_, and it exceeded that of other forest vegetation types significantly (*p* < 0.05). In the t_1_ stage, EC followed the order CB > DB > SF > CF, and EC in CB was significantly higher than in the other stand types (*p* < 0.05). For pH, DB was significantly higher than the other stand types in both the t_1_ and t_2_ stages (*p* < 0.05).

Regarding C content, CB and CF at stage t_1_ had significantly higher C contents than SF and DB (*p* < 0.05). At stage t_2_, the C content of CB was the lowest and was significantly lower than that of the other stand types (*p* < 0.05). In terms of N, at stage t_1_ the values followed the order CF > DB > CB > SF, with CF exhibiting a significantly higher N content than the other stand types (*p* < 0.05). At stage t_2_, N content in CB was significantly higher than in SF (*p* < 0.05). For P content, CB showed significantly greater values than SF and DB at both t_1_ and t_2_ (*p* < 0.05). At the t_1_ stage, CF exhibited the highest Li content, which was significantly greater than that of the other stand types (*p* < 0.05). During the t_2_ stage, Li content in SF was highest and was significantly greater than in the forest vegetation types (*p* < 0.05). For Ce content in the t_1_ stage, the ranking from highest to lowest was CF, DB, CB, and SF. The Ce content in CF was significantly higher than in other stand types (*p* < 0.05). In the t_2_ stage, CF showed the highest Ce concentration, averaging 0.1302 g·kg^−1^, and remained significantly greater than that of the other stand types (*p* < 0.05). During the t_1_ stage, He content in DB and CB was significantly higher than in SF and CF (*p* < 0.05). During the t_2_ stage, He content in SF was significantly higher than in the forest vegetation types (*p* < 0.05).

### 3.2. Composition of Litter-Inhabiting Fungal Communities in Different Stand Types

**Figure 3:**
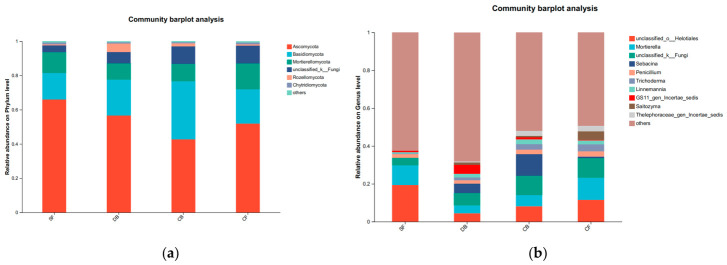
Community composition of litter-inhabiting fungal communities at the phylum (**a**) and genus (**b**) levels in different stand types at the t_1_ stage. SF (shrub forest), DB (deciduous broad-leaved forest), CB (coniferous and broad-leaved mixed forest), and CF (coniferous forest). In panel (**a**), the proportional representation of the major fungal phyla (relative abundance > 1%) is shown, with the remaining low-abundance phyla (≤1%) pooled as “Others”. In panel (**b**), the relative abundances of the top ten most abundant genera are displayed, while the remaining genera are combined into “Others”. Error bars represent standard deviation (SD); three replicates were assayed per stand type (*n* = 3).

Rarefaction curves for all samples approached saturation, and the Coverage index exceeded 97% across all samples, indicating that the sequencing depth was sufficient to capture the majority of fungal diversity and that the data reliably represent the community structure. High-throughput sequencing of the ITS gene from t_1_ stage litter yielded 924,672 valid sequences, which were taxonomically annotated using the RDP database. At the phylum level (Figure 3a), six phyla exceeded 1% relative abundance: Ascomycota, Basidiomycota, Mortierellomycota, unclassified_k__Fungi, Rozellomycota, and Chytridiomycota. Ascomycota and Basidiomycota together accounted for an average of 76.87%, making them the dominant fungal phyla in the t_1_ litter stage. Figure 3b displays the top 10 fungal genera by relative abundance, selected from 38 genera with relative abundance > 1%. The dominant genera included *unclassified_o__Helotiales*, *Mortierella*, and *unclassified_k__Fungi*. The “Other” category comprised the remaining genera with abundance > 1% and all genera with abundance ≤ 1%. Specifically, the relative abundance of Ascomycota in SF was significantly higher than in the other forest stand types (*p* < 0.05). The relative abundance of Basidiomycota in the CB was significantly higher than in the other stand types (*p* < 0.05). The relative abundance of unclassified_o__Helotiales in the SF was significantly higher than in the other stand types (*p* < 0.05).

**Figure 4:**
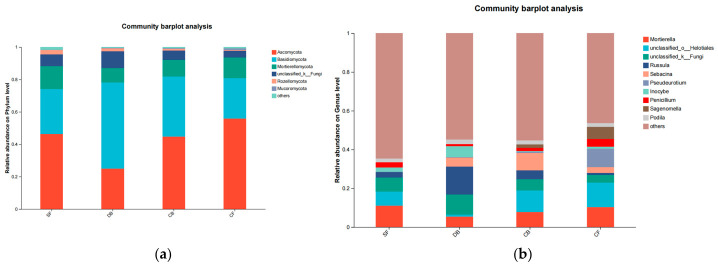
Community composition of litter-inhabiting fungal communities at the phylum (**a**) and genus (**b**) levels in different stand types at the t_2_ stage. SF (shrub forest), DB (deciduous broad-leaved forest), CB (coniferous and broad-leaved mixed forest), and CF (coniferous forest). In panel (**a**), the proportional representation of the major fungal phyla (relative abundance > 1%) is shown, with the remaining low-abundance phyla (≤1%) pooled as “Others”. In panel (**b**), the relative abundances of the top ten most abundant genera are displayed, while the remaining genera are combined into “Others”. Error bars represent standard deviation (SD); three replicates were assayed per stand type (*n* = 3).

High-throughput sequencing of fungal ITS regions in t_2_ stage litter yielded 980,638 valid sequences, which were taxonomically annotated using the RDP database. At the phylum level (Figure 4a), six phyla exceeded 1% relative abundance: Ascomycota, Basidiomycota, Mortierellomycota, unclassified_k__Fungi, Rozellomycota, and Mucoromycota. At the t_2_ litter stage, Ascomycota and Basidiomycota together accounted for an average relative abundance of 78.50%, making them the dominant fungal groups. Figure 4b presents the top 10 fungal genera by relative abundance, selected from 49 genera with relative abundances >1%. The dominant genera were *Mortierella*, *unclassified_o__Helotiales*, and *unclassified_k__Fungi*. The “Others” category comprised the remaining genera with abundance > 1% and all genera with abundance ≤ 1%. Notably, the relative abundance of Ascomycota in CF was significantly higher than in the other stand types (*p* < 0.05). The relative abundance of Basidiomycota in DB was significantly higher than in the other stand types (*p* < 0.05). The relative abundance of *Mortierella* in SF was significantly higher than in the other stand types (*p* < 0.05).

**Figure 5:**
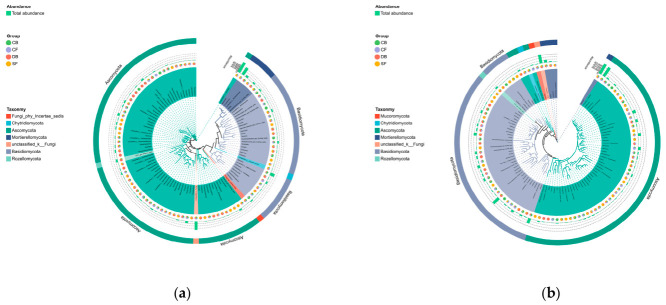
Phylogenetic evolutionary trees of litter-inhabiting fungal communities in different stand types. (**a**) Stage t_1_; (**b**) Stage t_2_. Note: Maximum likelihood (ML) method was used. Taxonomic labels follow standardized nomenclature: unclassified taxa are denoted with their highest resolved taxonomic rank prefix (k__ = kingdom, p__ = phylum, c__ = class, o__ = order, f__ = family, g__ = genus). For example, ‘unclassified_k__Fungi’ indicates sequences assigned to the kingdom Fungi but unclassified at lower taxonomic levels.

To visualize the taxonomic distribution and phylogenetic clustering of dominant fungal genera across different stand types, we aligned representative sequences from the top 100 genera (Figure 5). The phylogenetic analysis showed that these top 100 fungal genera across the two stages spanned 7 phyla, with the vast majority classified within Ascomycota and Basidiomycota. Furthermore, the analysis revealed that closely related taxa within these two dominant phyla exhibited non-random clustering patterns across stand types, suggesting that specific evolutionary lineages were directionally selected by stand-specific substrate chemistry.

### 3.3. Diversity of Litter-Inhabiting Fungal Communities in Different Stand Types

Alpha diversity indices were compared among stand types using One-way ANOVA with Tukey’s HSD post hoc test (α = 0.05). As shown in Table 2, fungi in DB during the t_1_ stage exhibited the greatest community diversity and species evenness among the four forest stand types, with both the Shannon index and Pielou-e evenness index reaching their highest values. Coverage indices for all four stand types exceeded 99%, indicating that the sequencing results reliably represent the fungal community composition at stage t_1_. The sequencing depth was sufficient to capture most microorganisms, including rare species, and thus faithfully reflected the litter-inhabiting fungal community status.

As shown in Table 3, the OTUs and Chao1 indices of CB were significantly higher than those of the other stand types (*p* < 0.05), indicating that CB maintained a relatively high fungal species richness and community abundance at stage t_2_. Coverage indices for all four stand types exceeded 99%, indicating that the sequencing results accurately represent the fungal community composition at the t_2_ stage. Sequencing depth was sufficient to capture most microorganisms, including rare species, and thus reliably reflected the litter-inhabiting fungal community.

**Figure 6:**
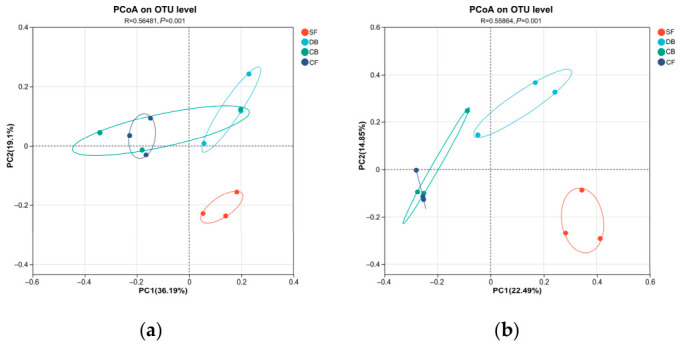
Beta diversity of litter-inhabiting fungi in different forest stand types. (**a**) Stage t_1_; (**b**) Stage t_2_.

Figure 6 showed that, during stages t_1_ and t_2_, the SF fungal community was relatively distant from the fungal communities of the other stand types, indicating a markedly different community structure in SF. In t_1_ stage, the shorter distances between CB and the DB and CF communities indicated relatively small differences in their fungal community structures. In t_2_ stage, the short distance between CB and CF again indicated a relatively small difference in their fungal community structures. The confidence ellipses of different stand types partially overlapped, but their positions differed markedly, reflecting variation in community composition among stand types. In the t_1_ stage, the CB samples showed a relatively large confidence ellipse, whereas the other groups had smaller ellipses, indicating pronounced structural heterogeneity within the CB group. In the t_2_ stage, the DB samples displayed a larger confidence ellipse than the other groups, indicating significant structural heterogeneity within the DB group.

### 3.4. Co-Occurrence Network Patterns of Litter-Inhabiting Fungal Communities in Different Stand Types

**Figure 7:**
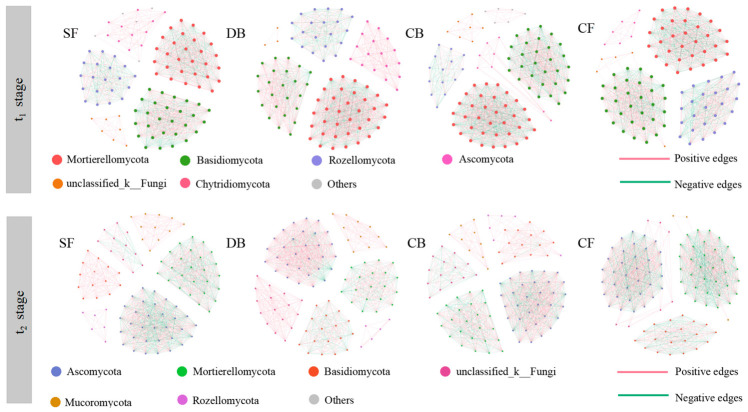
ASV-level co-occurrence networks of litter microbial communities across stand types at two time points (t_1_ and t_2_). Note: Networks were generated using Spearman correlations among the top 100 ASVs, applying significance cutoffs of |r| > 0.6 and FDR-corrected *p* < 0.05. Node size reflects relative abundance; red and green edges indicate positive and negative correlations, respectively. All groups had three replicates (*n* = 3).

Within the litter-inhabiting fungal community, the core microbiome comprised the 100 most abundant ASVs from the entire sample set. Based on these selected ASVs, a full co-occurrence network was generated to represent fungal interrelationships (Figure 7). The results revealed distinct topological patterns in the co-occurrence networks of litter-inhabiting fungi across stand types. The descriptive topology (Table 4) indicates that the number of nodes in the four plots at stages t_1_ and t_2_ is essentially the same. However, CF exhibits the highest total number of connections, average degree, and average clustering coefficient in both stages, while DB shows the longest average path length in both stages. In the co-occurrence networks of the four stand types, the proportion of positive correlations exceeded that of negative correlations. In contrast, the empirical networks (i.e., the microbial co-occurrence networks constructed in this study) show relatively high modularity, ranging from 0.598 to 0.691, which indicates significant modular structure.

**Figure 8:**
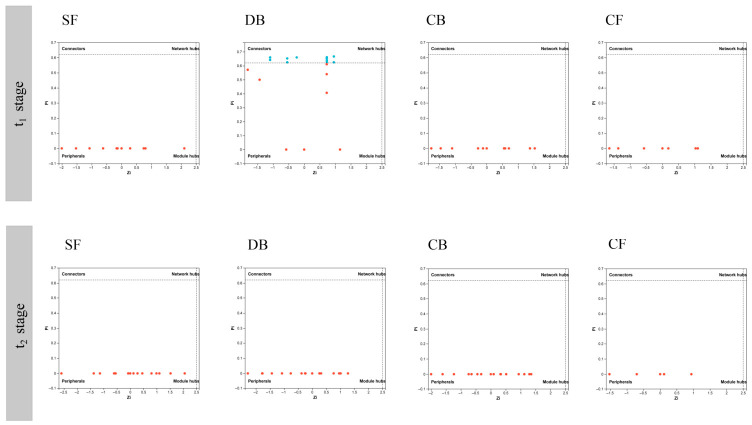
Zi–Pi analysis of microbial communities in litters of different stand types at stages t_1_ and t_2_. Note: Nodes in the plot represent individual fungal taxa. Based on their topological roles, nodes are classified into four categories: module hubs (nodes with high within-module connectivity; Zi > 2.5 and Pi < 0.62), connectors (nodes with high between-module connectivity; Zi < 2.5 and Pi > 0.62), network hubs (nodes with high connectivity both within and between modules; Zi > 2.5 and Pi > 0.62), and peripherals (nodes with low connectivity both within and between modules; Zi < 2.5 and Pi < 0.62). The first three categories are typically considered keystone taxa. Nodes are colored according to their topological roles: blue for connectors, and red for peripherals. This classification helps identify the distinct topological roles and keystone taxa within the co-occurrence networks.

Nodes were classified as network hubs, peripheral nodes, module hubs, or connectors based on their between-module connectivity (Pi) and within-module connectivity (Zi) values (Figure 8), since hubs, connectors, and module hubs are regarded as key taxa. Network hubs, connectors, and module hubs were identified as key taxa. The key taxa detected by DB at the t_1_ stage included Ascomycota, Basidiomycota, and unclassified_k__Fungi.

### 3.5. Differential Analysis of Litter-Inhabiting Fungal Communities in Different Stand Types

**Figure 9:**
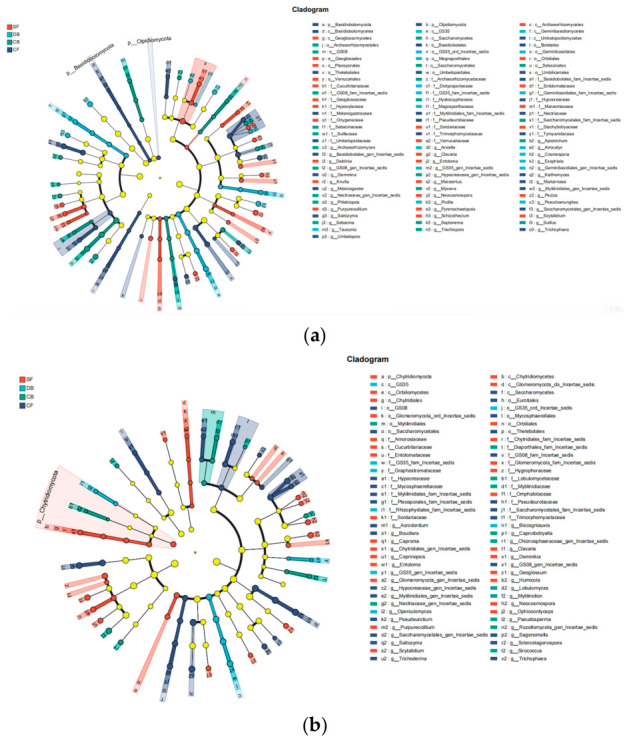
Evolutionary branching diagram of litter-inhabiting fungi in different stand types identified by LEfSe analysis. (**a**) Stage t_1_; (**b**) Stage t_2_. Note: Differential taxa were identified using LEfSe analysis with Kruskal–Wallis test (α = 0.05). Sample size: *n* = 3 per stand type.

**Figure 10:**
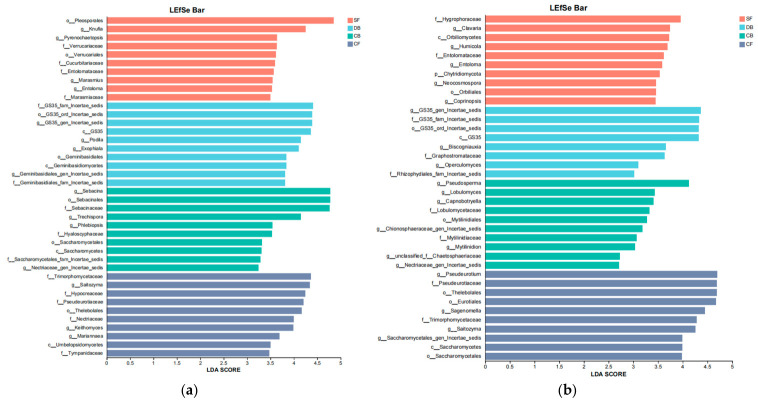
Bar chart of LDA value distribution of litter-inhabiting fungi in different stand types. (**a**) Stage t_1_; (**b**) Stage t_2_. Note: LDA score threshold = 2.0. Sample size: *n* = 3 per stand type.

We applied the linear discriminant analysis effect size (LEfSe) method to identify statistically significant fungal communities among the four stand types and to determine the core fungal communities at the t_1_ and t_2_ stages (Figure 9). The figure shows taxa with significant differences across five taxonomic levels, from phylum to genus. LEfSe analysis identified 40 fungal clades at the t_1_ stage (0 phyla, 4 classes, 7 orders, 14 families, 15 genera) and 38 fungal clades at the t_2_ stage (1 phylum, 3 classes, 6 orders, 9 families, 19 genera). Linear discriminant analysis (LDA) results (Figure 10) showed that the numbers of fungal taxa in the four stand types were 18, 10, 13, and 15 at stage t_1_ and 20, 3, 8, and 13 at stage t_2_. These results indicate that fungal composition in litter differs among stand types, with DB exhibiting the fewest biomarkers.

### 3.6. Functional Prediction of Litter-Inhabiting Fungal Communities in Different Stand Types

**Figure 11:**
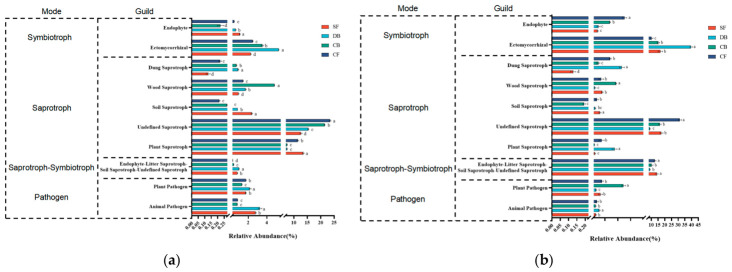
Secondary metabolism of litter-inhabiting fungi in different stand types (only the top 10 pathways with relative abundance > 1% are shown). (**a**) Stage t_1_; (**b**) Stage t_2_. Note: Different lowercase letters (a, b, c, d) above bars indicate significant between-group differences for each metabolic pathway (one-way ANOVA with Tukey’s HSD, *p* < 0.05). Error bars show SD; *n* = 3 per stand type.

FunGuild was used to predict litter-inhabiting fungal functions (Figure 11). The results identified four primary metabolic categories across the four stand types: Symbiotroph, Saprotroph, Saprotroph–Symbiotroph, and Pathotroph. Saprotroph constituted the primary function of the litter-inhabiting fungal community, representing more than 79% and 39% of metabolic pathways in stages t_1_ and t_2_, respectively. There were 26 and 25 secondary metabolic functions with relative abundances greater than 1% in the two stages, respectively. In both stages, the relative abundances of Ectomycorrhizal, Dung Saprotroph, and Animal Pathogen in DB were significantly higher than those in the other stand types (*p* < 0.05). The relative abundance of Wood Saprotroph was significantly higher in CB than in the other stand types (*p* < 0.05). The relative abundance of Soil Saprotroph peaked in SF and was significantly greater than in forest vegetation (*p* < 0.05). In Undefined Saprotroph, relative abundance was highest in CF and was significantly greater than in the other stand types (*p* < 0.05). In the t_1_ stage, the relative abundances of Endophyte and Plant Saprotroph were highest in SF and were significantly greater than those in forest vegetation (*p* < 0.05). The relative abundances of Endophyte, Litter Saprotroph, Soil Saprotroph, Undefined Saprotroph, and Plant Pathogen in DB were significantly higher than in the other stand types (*p* < 0.05). In the t_2_ stage, the relative abundance of Endophyte in CF was significantly higher than in the other stand types (*p* < 0.05). The relative abundance of Plant Saprotroph was highest in DB and was significantly greater than in stand types (*p* < 0.05). The relative abundances of Endophyte–Litter Saprotroph–Soil Saprotroph–Undefined Saprotroph were highest in SF and were significantly greater than those in CB and DB (*p* < 0.05). The relative abundance of Plant Pathogen was significantly greater in CB than in the other stand types (*p* < 0.05).

### 3.7. Analysis of the Correlation Between Litter Physical and Chemical Properties and Fungal Community Composition and Diversity of Different Stand Types

**Figure 12:**
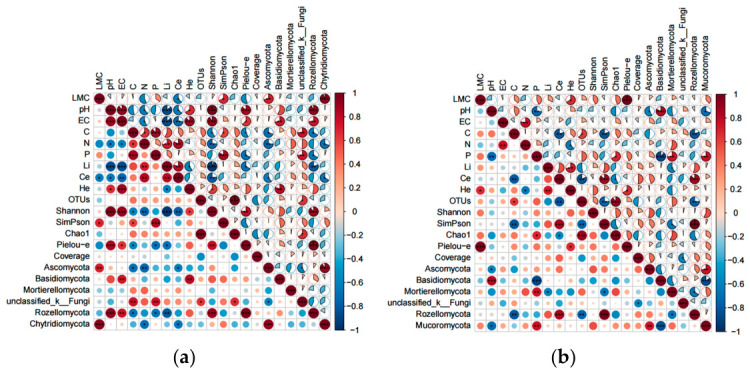
Correlation analysis between the physical and chemical properties of litter and the community composition and diversity of fungal phyla. (**a**) Stage t_1_; (**b**) Stage t_2_. Note: Correlations were calculated using Pearson correlation coefficients with FDR correction for multiple testing: *** *p* < 0.001; ** *p* < 0.01; * *p* < 0.05; *n* = 12 samples.

Litter physicochemical properties correlated significantly with fungal community composition and diversity (Figure 12). At the t_1_ stage, EC and He were both strongly positively correlated with Basidiomycota (*p* < 0.01). Rozellomycota showed a strong positive correlation with pH and EC (*p* < 0.01) and a significant negative correlation with C, P, and Li (*p* < 0.05). Chytridiomycota showed a strong positive correlation with LMC (*p* < 0.01) and a significant negative correlation with N and Ce (*p* < 0.05). Ascomycota showed a significant negative correlation with C, N, and Ce (*p* < 0.05). Both C and P were highly significantly positively correlated with unclassified_k__Fungi (*p* < 0.01). In the t_2_ stage, Basidiomycota exhibited a highly significant positive correlation with pH (*p* < 0.01) and a highly significant negative correlation with P (*p* < 0.01). pH was significantly and negatively correlated with the relative abundance of Ascomycota (*p* < 0.05). Mucoromycota exhibited a highly significant positive correlation with P (*p* < 0.01) and a significant negative correlation with pH (*p* < 0.05). Mortierellomycota correlated strongly and positively with P (*p* < 0.01) and significantly negatively with Li (*p* < 0.05). Rozellomycota correlated strongly and positively with Ce (*p* < 0.01) and strongly negatively with C (*p* < 0.01). At the t_1_ stage, the Simpson index correlated positively and highly significantly with P (*p* < 0.01) and showed significant positive correlations with LMC and C (*p* < 0.05). The Shannon index showed positive correlations with pH, EC, and He (*p* < 0.05) and negative correlations with N, Li, Ce, and C (*p* < 0.05). The Pielou-e index showed positive correlations with pH and EC (*p* < 0.05) and negative correlations with C, P, and Li (*p* < 0.05). At the t_2_ stage, the OTUs index showed a significant positive correlation with C (*p* < 0.05) and a highly significant negative correlation with Ce (*p* < 0.01). The Simpson index exhibited a highly significant positive correlation with Ce (*p* < 0.01) and a highly significant negative correlation with C (*p* < 0.01). The Pielou-e index correlated positively and strongly with LMC (*p* < 0.01) and positively with He (*p* < 0.05). The Chao1 index correlated positively with P (*p* < 0.05) and negatively with Ce (*p* < 0.05).

**Figure 13:**
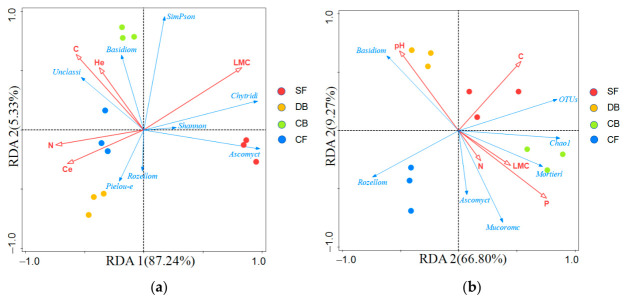
Redundancy analysis (RDA) of litter-inhabiting fungal communities, diversity, and environmental variables at the phylum level. (**a**) Stage t_1_; (**b**) Stage t_2_. Note: RDA ordination of litter-inhabiting fungal communities (phylum level), diversity, and environmental factors (*n* = 12). Forward selection with 999 permutations (FDR; *p* < 0.05) was applied to identify significant predictors. Red arrows = environmental variables; blue arrows = fungal phyla/diversity indices; circles = samples.

Prior to redundancy analysis (RDA), detrended correspondence analysis (DCA) was performed to evaluate the gradient length of the species data. The results explicitly showed that the length of the first DCA axis was 0.48 at stage t_1_ and 0.19 at stage t_2_. Since both values were well below the threshold of 3.0 standard deviations (SD), a linear response model (RDA) was deemed highly appropriate for analyzing the relationships between fungal communities and environmental variables. We analyzed relationships among litter physicochemical properties, stand types, and fungal communities by treating fungal phyla and diversity as response variables and litter physicochemical properties as explanatory variables (Figure 13). In the t_1_ stage, RDA1 accounted for 87.24% of the variation and RDA2 for 5.33% (Figure 13a). In the t_2_ stage, RDA1 accounted for 66.80% of the variation and RDA2 for 9.27% (Figure 13b). LMC and C explained the most variation in litter-inhabiting fungal community structure and diversity at the t_1_ and t_2_ stages (R^2^ > 0.90, *p* < 0.01).

### 3.8. Correlation Between Litter Physical and Chemical Properties and Fungal Community Function in Different Stand Types

**Figure 14:**
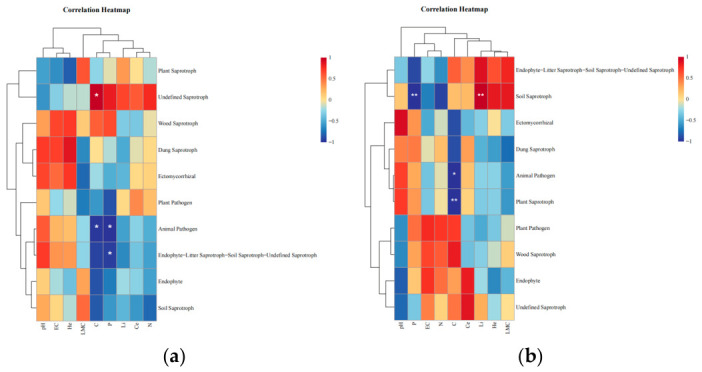
Correlation between the physical and chemical properties of litter and the relative functional abundance of different stand types (only the top 10 functions with relative abundance > 1% are shown). (**a**) Stage t_1_; (**b**) Stage t_2_. Note: Correlations were calculated using Pearson correlation coefficients with FDR correction for multiple testing: ** *p* < 0.01; * *p* < 0.05. Sample size: *n* = 12 samples (3 replicates × 4 stand types). Error bars represent standard deviation (SD).

The metabolic functions of the litter-inhabiting fungal community displayed distinct correlations with physicochemical properties (Figure 14). In stage t_1_, C correlated positively with Undefined Saprotroph (*p* < 0.05) and negatively with Animal Pathogen (*p* < 0.05). P was significantly negatively correlated with Animal Pathogen, Endophyte—Litter Saprotroph—Soil Saprotroph—Undefined Saprotroph (*p* < 0.05). In the t_2_ stage, Soil Saprotroph exhibited a highly significant positive correlation with Li (*p* < 0.01) and a highly significant negative correlation with P (*p* < 0.01). C showed a highly significant negative correlation with Plant Saprotroph (*p* < 0.01) and a significant negative correlation with Animal Pathogen (*p* < 0.05).

## 4. Discussion

### 4.1. Litter Physicochemical Properties Across Stand Types

The dynamic evolution of litter’s physical and chemical properties forms the primary substrate basis that drives microbial decomposition and directly determines the organic matter mineralization pathway and element turnover efficiency [30,31]. This study showed that, from stage t_1_ to t_2_, the physical and chemical characteristics of litter in different forest stands exhibit pronounced stage-specific differentiation and forest-type specificity. The LMC of SF was significantly higher than that of forest types at both stages, which primarily reflects the SF’s loose initial structure and high porosity that create abundant micropore networks capable of adsorbing and retaining moisture [32]. This physical property allows SF litter to sustain a relatively high water-holding capacity even during the late decomposition stage. During the t_1_ stage, EC peaked in CB, reflecting rapid leaching and release of readily soluble organic matter and mineral ions from the mixed substrate in the initial phase [33]. DB maintained a significantly higher pH throughout decomposition, driven by the high ash content of broad-leaved litter, the large release of alkaline cations, and the slow mineralization of organic acids, which together formed a strong acid–base buffer in the microenvironment [34].

Nutrient dynamics further support the influence of substrate quality on the decomposition trajectory. During the t_1_ stage, C contents in both CB and CF remained relatively high, primarily because the coniferous components are rich in structural polysaccharides and secondary metabolites. In this initial phase, physical leaching was limited and microbial assimilation rates were low, so much structural carbon remained in the substrate and the overall carbon pool was not rapidly mineralized or consumed [35]. By the t_2_ stage, CB’s carbon content reached its minimum, which we attribute to a non-additive effect of the mixed substrate: readily available carbon released by broad-leaved components relieved microbial nutrient limitation and thus triggered co-metabolism of coniferous structural carbon, accelerating mineralization and depletion of the overall carbon pool [36]. This non-additive acceleration aligns with the findings of Hättenschwiler et al. [37] in diverse plant litter mixtures, where complementary litter traits synergistically overcome nutrient limitations. In contrast, CB maintained relatively high nitrogen and phosphorus retention at stage t_2_, mainly because the complementary nutrients of coniferous and broad-leaved substrates produce distinct biochemical effects. Labile carbon from broad-leaved litter drives temporary microbial immobilization of nutrients, while polyphenols leached from conifers complex with nitrogen and, together with volcanic mineral micro-regions, stabilize phosphorus. These processes reduce late-stage leaching and give the mixed substrate a stronger potential for slow nutrient release [38]. The evolution of structural components underscores a persistent constraint on anti-decomposition traits. Li and Ce of CF remained high throughout the process, likely reflecting the inherently lignified structure and phenolic chemical defenses of coniferous substrates [39]. The persistent retention of these recalcitrant components in CF is consistent with observations in other coniferous ecosystems, where high lignin content imposes strong substrate-selection pressure and delays late-stage mineralization [40]. The spatial barrier formed by lignin and associated phenolic compounds markedly reduces microbial decomposition of structural carbon, causing persistent retention of recalcitrant components and thereby imposing substrate-selection pressure that favors succession of late-stage obligate lignocellulose-degrading groups [41]. During the t_2_ stage of SF, Li and He increased noticeably. This rise likely reflects rapid decomposition of SF, which as pioneer vegetation on the lava plateau decomposes relatively quickly during the early stage. Many labile components are rapidly mineralized during early decomposition, while more refractory constituents become relatively enriched at later stages [42]. In summary, litter physical and chemical properties interact and reciprocally influence one another across stand types and succession stages. Through complex biogeochemical cycling processes, they establish the environmental drivers for subsequent analyses of community reconstruction and functional differentiation.

### 4.2. Fungal Community Composition and Diversity

Previous studies have shown that volcanic eruptions can alter microbial community structure [43]. Dominant microbial taxa and forest stand type are key factors shaping microbial community composition and function [44]. The results showed that at the phylum level Ascomycota and Basidiomycota remain dominant in both the t_1_ and t_2_ stages (average relative abundance >76%). The observed fungal community dynamics followed a defined pattern shaped jointly by stand-specific substrate filtering and deterministic successional shifts. This pattern matches observations from global forest ecosystems and reflects fungal adaptive strategies to litter substrates across different stand types [45]. Tao Huiyun et al. [46] noted that Ascomycota is dominated by saprophytic fungi that secrete diverse extracellular enzymes to degrade recalcitrant organic matter, whereas Basidiomycota contains many lignin-degrading species that play a key role in decomposing Li and Ce. To provide specific examples, classic ascomycetous genera such as Penicillium and Aspergillus are renowned for secreting hydrolytic enzymes (e.g., cellulases and hemicellulases) to efficiently break down polysaccharides [47]. Conversely, ligninolytic basidiomycetous genera, particularly white-rot fungi like Trametes and Phanerochaete, primarily produce oxidative enzymes (e.g., laccase and manganese peroxidase) that catalyze the depolymerization of complex lignin structures [48].

Regarding the differences among stand types (spatial/substrate filtering), the distinct community structures align with the “habitat filtering” hypothesis, where specific litter chemistry selects for specialized taxa. For Ascomycota, at the t_1_ stage, SF exhibited the highest relative abundance, a pattern primarily driven by the combined effect of high LMC and low structural carbon components in SF litter [32]. This microhabitat, characterized by “low physical barrier and high water availability,” lowers substrate resistance to decomposition and accelerates the early release of soluble nutrients, thereby creating favorable colonization conditions for Ascomycota species that follow r-strategist growth strategies [49]. By stage t_2_, the further rise in Ascomycota abundance in CF likely reflects carbon–nitrogen limitation and chemical stress from residual phenolic compounds and resin acids left after selective decomposition of the CF substrate [50]. The succession trajectory of Basidiomycota may reflect coordinated regulation between the availability of the structural carbon pool and the microenvironment’s physicochemical conditions. In the t_1_ stage, its early enrichment in CB likely resulted from a relatively large initial structural carbon pool combined with the highest P content in CB litter. Our results suggest that phosphorus availability may relieve the nutrient constraint on oxidase synthesis and potentially prime the cometabolic network and lignin-degrading enzyme system in basidiomycetes. By t_2_, Basidiomycota reached peak abundance in DB, primarily because DB litter maintains the highest pH throughout the process. An alkaline microenvironment may favor the catalytic activity of oxidases such as laccase and manganese peroxidase. In addition, broad-leaved substrates lack the strong chemical defenses characteristic of conifers, and their residual lignin–carbohydrate complexes provide highly enzyme-accessible substrates for obligate lignin-degrading groups [51,52]. Phylogenetic analysis of evolutionary trees further supports the above differentiation. During the t_1_ and t_2_ stages, the top 100 dominant genera clustered tightly within the core branches of Ascomycota and Basidiomycota, and the distributions of closely related taxa were highly consistent across forest stands. The results showed that chemical evolution trajectories of litter in different forest stands do not recruit microorganisms at random. Instead, strong environmental filtering directionally enriches conservative taxa with matching metabolic profiles, thereby dictating a predictable succession of community composition along the substrate quality gradient. Regarding the differences between decomposition stages (temporal/successional shifts), the transition from a community dominated by “phyllosphere-associated” taxa to one enriched with “soil-associated” taxa represents a clear deterministic pattern across all stand types. In the early stage of decomposition, colonizing fungi, such as some Ascomycota, likely originate from or share functional traits with the phyllosphere microbial community carried on the leaves. They possess a broad-spectrum hydrolase system and rapidly exploit readily degradable carbon sources. In the late stage of decomposition, as litter and the soil matrix become more closely integrated and recalcitrant components accumulate, the proportion of soil-derived taxa (for example, certain Basidiomycota and Zygomycota) in the community increased markedly, reflecting a microhabitat shift from the “plant residue surface” to the “soil–litter interface” [53].

For alpha diversity, the Shannon index and Pielou-e index of DB at the t_1_ stage were highest. This pattern primarily reflects the moderate structural complexity of DB litter at the initial stage and its relatively high pH microenvironment, which together expanded niche breadth, reduced early interspecific competition, and thus permitted coexistence of broad-spectrum saprotrophic fungi and pioneer colonizers [54]. By stage t_2_, most diversity indices converged, yet CB retained the highest OTUs and Chao1 index. This persistence likely reflects asynchronous decomposition of coniferous and broad-leaved components: labile carbon released from broad-leaved trees and recalcitrant lignin from conifers create chemically heterogeneous microdomains. Resource pulses and substrate diversity delayed the monopolization by a single dominant group, thereby preserving a relatively large potential species pool [55]. The subsequent phased reorganization of the beta diversity pattern further supports the inferred trajectory of environmental filtering. In this study, fungal communities in SF litter at the t_1_ and t_2_ stages were clearly distinct from those in other stand types, primarily because SF, as a pioneer species, creates a unique chemical environment. The SF litter’s composition—high in wax and low in lignin—contrasts sharply with that of mature forest vegetation [56]. Internal variability of CB was highest at the t_1_ stage, reflecting the complex carbon-source gradient created by mixed-litter inputs, which provides diverse niches for different functional groups [57]. In the t_2_ stage, DB showed maximal internal variability. This likely reflects that, in the late decomposition of DB litter, the remaining components are more sensitive to microenvironmental fluctuations. In summary, the dynamic evolution of fungal diversity is governed by the synergistic effects of substrate chemical characteristics, micro-domain heterogeneity, and stage-specific environmental filtering, which together clarify the mechanisms by which microbial communities adaptively assemble during litter decomposition.

### 4.3. Co-Occurrence Network Topology and Biomarker Analysis

To elucidate the relationship between stand type and fungal community structure, we performed network analysis. The results indicated that fungal communities in CF litter at both stages exhibited greater connectivity than those in DB, as reflected by higher average clustering coefficients and mean degrees. With the same number of nodes, CF exhibited more connections, a higher average degree, and a greater average clustering coefficient than the other stand types, consistent with previous findings and indicating that the fungal community structure in CF litter is more complex and stable than in the other stand types. This difference in network structure further confirms that stand type shapes the organization of litter-inhabiting fungal communities [58]. Interestingly, the higher network connectivity in CF under chemical stress (high phenolics) contrasts with the general expectation that environmental stress simplifies microbial networks; this aligns with recent findings suggesting that complex, recalcitrant substrates foster cooperative interactions among fungi to overcome decomposition barriers [59]. Keystone species denote taxonomic units that are highly connected and exert a disproportionate influence on the community structure [59]. Research shows that keystone species in natural ecosystems often change during succession. These shifts substantially influence biogeochemical cycles of carbon, nitrogen, and phosphorus and thereby regulate ecosystem function [60,61]. Key species also differ across stand types, with those in the DB litter being most abundant. This pattern suggests that DB traits substantially influence the litter microbial community.

Biomarker analysis further provides important evidence. Biomarkers are fungal taxa that are significantly enriched in the litter of particular stand types and thus reflect environmental differences [62]. Notably, in the CB, the coexistence of coniferous and broad-leaved plants produces substantial differences in root exudates and litter properties between the two plant types. This diversification of root exudates and of nutrient substrates in the litter matrix suppresses the dominance of any single fungal group. The suppression of single-taxon dominance in CB is also consistent with the “portfolio effect” observed in diverse litter mixtures by other researchers, where high substrate heterogeneity prevents competitive exclusion by a single functional group [37]. Consequently, the LEfSe analysis comparing the two stages identified only a relatively small number of significantly different taxa (i.e., biomarkers) [63,64]. This finding aligns with the network analysis results and together they reveal the comprehensive mechanisms by which stand types shape the structure and function of litter-inhabiting fungal communities. Please note that while *n* = 3 per stand type is the minimum standard for basic statistical comparisons and precludes the application of formal bootstrap or permutation tests for network metrics due to limited statistical power, the observed topological differences are not merely descriptive. The robustness of our network inference is strongly supported by high consistency with independent, statistically robust analyses. For instance, the higher network connectivity and modularity observed in CF align perfectly with its significantly higher lignin content (*p* < 0.05) and specific LEfSe-identified biomarkers. This cross-validation ensures that the identified network structures reliably reflect genuine ecological responses to substrate chemistry rather than random sampling artifacts.

### 4.4. Predicted Functional Potential of Fungal Communities

The evolution of nutritional functional types and secondary metabolic potential in the litter-inhabiting fungal community on the Jingpo Lake lava plateau exhibited pronounced stage-related and stand-specific patterns, which were primarily co-shaped by succession in substrate chemical composition and by the physicochemical stresses of the microenvironment [65]. In primary trophic modes, saprotrophs dominated at t_1_ (>79%) and declined markedly by t_2_ (>39%). This shift does not indicate functional loss but instead reflects a change in decomposition strategy from rapid mineralization of labile carbon to selective utilization of recalcitrant components, consistent with previous reports [66]. A critical question arising from these pronounced compositional shifts is whether ecological functions are maintained. Our results strongly suggest the presence of functional redundancy among the fungal groups. Although the relative abundances of specific taxa and trophic modes shifted markedly from t_1_ to t_2_ (e.g., from Plant Saprotrophs to Soil Saprotrophs and Endophytes), the overall metabolic capacity for litter decomposition was sustained. This taxonomic turnover represents a “functional handover” rather than a loss of function: while early-stage fast-growing taxa rapidly mineralize labile carbon, late-stage specialized degraders seamlessly take over the breakdown of recalcitrant compounds. Such functional redundancy ensures that ecosystem-level processes remain stable despite severe environmental filtering and compositional changes, a phenomenon widely observed during fungal successional dynamics [40]. As soluble nutrients are depleted, the fungal community progressively shifts toward symbiotic and pathogenic lifestyles to cope with the nutritional scarcity and chemical stress of the late-stage substrate.

Secondary functional prediction further revealed that stand substrate characteristics directed the screening of fungal metabolic profiles. CB maintained a relatively high relative abundance of Wood Saprotroph throughout the process, a pattern mainly attributable to its litter’s initially high He content and to significant phosphorus availability. He supplied abundant primary carbon skeletons, and adequate phosphorus may have relieved energy and cofactor constraints during oxidase synthesis, thereby potentially promoting colonization and proliferation of obligate lignin-degrading taxa [67,68]. By stage t_2_, the marked enrichment of Plant Pathogen in CB likely reflects substrate microenvironment deterioration following depletion of readily available carbon, opportunistic colonization driven by accumulated secondary metabolites, and an ensuing imbalance in interspecific competition. In the t_1_ stage of SF, Soil Saprotroph and Plant Saprotroph dominated, reflecting the rapid microenvironmental turnover driven by high water-holding capacity and low structural resistance. In the t_2_ stage, it shifted towards Endophyte—Litter—Soil—Undefined Saprotroph, indicating that under the conditions of nutrient depletion and relative enrichment of structural components, the SF substrate promotes fungi to evolve a broad—spectrum metabolic strategy across habitats to maintain carbon acquisition [66]. Across the DB, Ectomycorrhizal, Animal Pathogen, and Plant Saprotroph groups occurred at relatively high levels, reflecting abundant nutrient inputs from litter and elevated plant diversity. These conditions fostered a complex microbial interaction network. This functional characteristic reflects the balance between mutualistic and parasitic interactions in the broad-leaved forest system and represents an adaptive response of the microbial community to high-quality litter substrates [69]. The continued dominance of Undefined Saprotroph in CF and Endophyte at the t_2_ stage indicates that the micro-domain, under high phenolic/resin acid stress, selects for fungi with conservative and flexible metabolic strategies: when lignin degradation is clearly inhibited, taxa capable of broad-spectrum substrate use or of tissue symbiosis prevail through functional redundancy to cope with nutrient scarcity [70]. Functional prediction results further support a shift in community origin and niche. At the t_1_ stage, fungal functional groups were dominated by typical Plant Saprotrophs, indicating rapid colonization and initial decomposition of fresh litter by phyllosphere- or surface-derived fungi. By the t_2_ stage, fungal functional groups had shifted markedly toward Soil Saprotrophs and Endophyte-Litter-Soil-Undefined Saprotrophs [71]. This shift in metabolic pathways indicates that the fungal community’s functional focus moved from simple plant tissue degradation to more complex soil organic matter turnover and cross-kingdom material cycling, completing a functional succession from ‘phyllosphere-associated’ to ‘soil-associated’. It is important to acknowledge the inherent limitations of the FUNGuild database approach. In our dataset, approximately 70% of the total ASVs were successfully assigned to specific trophic guilds (e.g., Saprotrophs, Pathotrophs), while the remaining ~30% of sequences were categorized as ‘unknown’ or remained unannotated. This unannotated fraction likely reflects the incomplete functional characterization of many fungal taxa in current databases, particularly for rare or understudied lineages common in volcanic ecosystems. Crucially, the dominant functional patterns (e.g., the overwhelming prevalence of Saprotrophs and their shift toward Soil Saprotrophs/Endophytes) were primarily driven by the most abundant ASVs, which were successfully annotated. This suggests that the observed functional succession trends reliably reflect the community’s metabolic potential. The unannotated portion, while substantial, is unlikely to alter the primary ecological conclusions regarding the dominance of saprotrophic strategies during decomposition. In summary, the functional potential of litter-inhabiting fungi is not static but is dynamically reprogrammed as decomposition progresses and the substrate chemistry of the forest stand evolves.

### 4.5. Environmental Drivers of Fungal Community Variation

The compositional succession of the litter-inhabiting fungal community is regulated synergistically, stage by stage, by multiple physical and chemical factors, and the core driving pathways shift systematically over the course of decomposition. In stage t_1_, water availability and the micro-domain ionic environment are the primary limiting factors shaping the community pattern. LMC correlated strongly with the relative abundance of Chytridiomycota, reflecting moisture’s essential role in spore germination and hyphal growth. Microenvironments with high water-holding capacity reduced the substrate’s physical resistance and supplied the liquid-phase conditions needed for extracellular enzyme diffusion and substrate contact, thereby promoting rapid colonization by early pioneer groups [70]. The rise in pH and EC correlated strongly with Basidiomycota and Rozellomycota, indicating that a neutral to mildly alkaline microenvironment combined with moderate ionic strength favored initial enzymatic activity and broadened resource-utilization niches [72]. In contrast, C, N, and structural components showed significant negative correlations with Ascomycota. This association did not arise from nutrient inhibition; instead, the substrate’s high structural complexity and anti-decomposition properties during early stages increased microbial colonization resistance and delayed the rapid expansion of broad-spectrum saprophytic taxa [71]. As easily degradable components became depleted, community assembly during the t_2_ stage shifted its driving focus to the chemical properties of the recalcitrant carbon pool and to key limiting nutrients. During this stage, Basidiomycota relative abundance correlated closely with pH, indicating that a slightly alkaline microenvironment stabilizes and enhances the catalytic activity of the lignin-degrading enzyme system. The strong positive responses of Mucoromycota and Mortierellomycota to P suggest the ecological regulatory role of phosphorus as a key cofactor in nucleic acid synthesis and energy metabolism, particularly under intensified nutrient limitation during the late decomposition stage [73]. In addition, the high C and Ce substrates altered substrate accessibility and thereby reshaped the competitive relationships among specific taxa, promoting a shift in the community toward tolerant taxa with obligate metabolic lineages.

The spatiotemporal dynamics of fungal community diversity are also governed by the sequential filtering of the physicochemical factors described above. During the t_1_ stage, increases in pH, EC, and He correlated significantly with the Shannon and Pielou-e indices. This likely reflects the combined effects of a relatively neutral microenvironment, moderate ionic strength, and abundant hemicellulose substrates, which broadened the nutritional niche and thereby mitigated interspecific competitive exclusion driven by early resource enrichment. C, N, Li, Ce, and P were negatively correlated with the diversity index, indicating that high structural barriers and nutrient retention during the initial stage constrained synchronous colonization by multiple taxa and preserved the primary heterogeneity of community composition [74]. Upon entering stage t_2_, diversity dynamics became strongly governed by the quality and physical structure of the carbon pool. In this stage, C content correlated negatively with the Simpson index and positively with the total number of OTUs, suggesting that the slow energy release from recalcitrant carbon reduced the monopolizing capacity of dominant taxa. This reduction allowed more species to coexist at lower relative abundances and thereby supported a larger potential species pool [75]. Ce showed a significant positive correlation with the Simpson index, suggesting that the high-cellulose matrix may provide a favorable resource space for taxa with specific degradation capabilities by constructing a complex physical network, thereby increasing the community’s overall dominance [76]. In addition, the synergy of LMC and He provided continuous moisture buffering and supplies a transitional carbon source for late-stage fungal metabolism, thereby alleviating environmental stress from substrate aging. This enabled fungi with different nutritional strategies to more evenly exploit microdomain resources, thus maintaining community evenness. RDA confirmed that LMC and C were the key factors regulating fungal community structure at the t_1_ and t_2_ stages, respectively, which agrees with previous findings [75,77].

The coupling between functional potential and physicochemical factors reveals how fungal metabolic strategies dynamically respond to substrate availability. In stage t_1_, C content correlated strongly with Undefined Saprotroph and showed a negative correlation with Animal Pathogen. This suggests that the relatively abundant carbon source in the initial stage supplies sufficient energy for the broad-spectrum saprophytic network, allowing rapid biomass accumulation that creates niche suppression and thereby inhibits colonization and expansion of opportunistic pathogenic taxa [78]. Meanwhile, the negative correlation between P content and Animal Pathogen and Endophyte—Litter—Soil—Undefined Saprotroph may indicate that, under limited phosphorus availability in early stages, fungi adopt a conservative nutrient-interception strategy to reduce reliance on metabolic pathways with high phosphorus demand [79]. Upon entering the t_2_ stage, reshaping of functional groups is driven mainly by accumulation of recalcitrant components and by imbalanced nutrient stoichiometry. Soil saprotroph abundance correlated positively with Li and negatively with P. Under resource-poor microdomains, microorganisms sustain basic carbon and nitrogen turnover by increasing broad-spectrum substrate uptake and favoring oligotrophic metabolic pathways. High lignin residues supply a persistent refractory carbon skeleton, while P limitation shifts metabolism toward more efficient nutrient cycling and thus promotes relative enrichment of the Soil Saprotroph functional pathway [78]. Furthermore, C content correlated significantly negatively with both Plant Saprotroph and Animal Pathogen, which confirms that depletion of readily available carbon in the late stage reduces the competitive advantage of parasitic and obligate plant-degradation strategies [80]. While bacteria are also crucial agents in litter decomposition, this study focused exclusively on fungi because their extensive mycelial networks and specialized enzymatic machinery make them the primary drivers of degrading recalcitrant polymers (e.g., lignin) in nutrient-limited volcanic habitats. Nevertheless, bacterial communities likely play a complementary role, particularly in the early mineralization of labile carbon and within the fungal hyphosphere [81]. Future multi-kingdom studies integrating bacterial profiling would further elucidate the holistic microbial interaction networks driving litter decomposition. In conclusion, this study preliminarily revealed the complex interactions between microbial metabolic functions and the substrate environment during litter decomposition in volcanic habitats by analyzing correlations between the relative abundances of fungal functional groups at different decomposition stages and the litter’s physicochemical properties.

## 5. Conclusions

This study aimed to elucidate the stage-specific differences in fungal community structure and functional potential during litter decomposition across different stand types on the Jingpo Lake lava plateau. Our findings reveal that Ascomycota and Basidiomycota dominate the litter-inhabiting fungal communities, which undergo a deterministic successional shift from early-stage “phyllosphere-associated” taxa driven by moisture availability to late-stage “soil-associated” taxa driven by recalcitrant carbon quality. Stand-specific substrate properties, particularly litter moisture content and carbon quality, act as the primary environmental filters shaping community assembly, network topology, and functional redundancy. Specifically, coniferous substrates foster more complex fungal co-occurrence networks, while mixed litter substrates suppress single-taxon dominance through high heterogeneity. Ultimately, this tightly coupled plant-litter-microorganism feedback loop drives multi-stage nutrient turnover and maintains ecosystem stability in nutrient-limited volcanic habitats. These insights provide a novel microbiological perspective on litter decomposition mechanisms in lava plateau ecosystems, offering a theoretical basis for targeted vegetation restoration, forest nutrient management, and the ecological recovery of fragile volcanic landscapes. Future research integrating multi-kingdom microbial profiling and high-frequency time-series monitoring will further unravel the holistic interaction networks driving these successional dynamics.

## Figures and Tables

**Table 1:** Dominant plant species (based on field survey criteria).

Vegetation Types	Dominant Species
SF	*Lespedeza davurica*, *Lonicera maackii*, *Acer tataricum* subsp. *ginnala*
DB	*Populus davidiana*, *Quercus mongolica*, *Betula platyphylla*
CB	*Populus davidiana*, *Quercus mongolica*, *Betula platyphylla*, *Pinus koraiensis*
CF	*Pinus koraiensis*

**Table 2:** Alpha diversity and abundance indices of litter-inhabiting fungi in different forest stand types at stage t_1_.

t_1_ Stage	OTUs	Shannon	SimPson	Chao1	Pielou-e	Coverage
SF	1105.67 ± 253.50 ^a^	6.50 ± 1.08 ^a^	0.95 ± 0.14 ^a^	1155.28 ± 246.48 ^a^	0.64 ± 0.14 ^a^	0.99 ± 0.01 ^a^
DB	1208.67 ± 146.84 ^a^	7.18 ± 0.14 ^a^	0.94 ± 0.01 ^a^	1309.34 ± 186.07 ^a^	0.73 ± 0.03 ^a^	0.99 ± 0.01 ^a^
CB	1270.00 ± 205.65 ^a^	6.82 ± 0.29 ^a^	0.96 ± 0.02 ^a^	1367.65 ± 232.59 ^a^	0.67 ± 0.02 ^a^	0.99 ± 0.01 ^a^
CF	1282.00 ± 123.50 ^a^	6.97 ± 0.51 ^a^	0.95 ± 0.02 ^a^	1364.32 ± 133.51 ^a^	0.69 ± 0.06 ^a^	0.99 ± 0.01 ^a^

Note: The first column in the table shows the type of sample site. SF (shrub forest), DB (deciduous broad-leaved forest), CB (coniferous and broad-leaved mixed forest), CF (coniferous forest). Different lowercase letters (a) within the same column indicate significant differences among the four stand types (one-way ANOVA followed by Tukey’s HSD test, *p* < 0.05).

**Table 3:** Alpha diversity and abundance indices of litter-inhabiting fungi in different forest stand types at stage t_2_.

t2 Stage	OTUs	Shannon	Simpson	Chao1	Pielou-e	Coverage
SF	791.67 ± 29.57 ^ab^	6.58 ± 0.35 ^a^	0.93 ± 0.02 ^a^	820.19 ± 47.01 ^ab^	0.69 ± 0.05 ^a^	0.99 ± 0.01 ^a^
DB	699.34 ± 73.76 ^b^	6.10 ± 0.47 ^a^	0.93 ± 0.03 ^a^	737.85 ± 76.38 ^b^	0.63 ± 0.08 ^a^	0.99 ± 0.01 ^a^
CB	915.67 ± 117.62 ^a^	6.36 ± 0.21 ^a^	0.93 ± 0.02 ^a^	946.07 ± 119.58 ^a^	0.64 ± 0.02 ^a^	0.99 ± 0.01 ^a^
CF	699.00 ± 79.00 ^b^	6.19 ± 0.37 ^a^	0.93 ± 0.02 ^a^	723.14 ± 96.28 ^b^	0.64 ± 0.05 ^a^	0.99 ± 0.01 ^a^

Note: The first column in the table shows the type of sample site. SF (shrub forest), DB (deciduous broad-leaved forest), CB (coniferous and broad-leaved mixed forest), CF (coniferous forest). Different lowercase letters (a, b) within the same column indicate significant differences among the four stand types (one-way ANOVA followed by Tukey’s HSD test, *p* < 0.05).

**Table 4:** Topological structure of the microbial co-occurrence network of litter in different forest stand types at stages t_1_ and t_2_.

Topological Features	t_1_ Stage				t_2_ Stage			
	SF	DB	CB	CF	SF	DB	CB	CF
Node number	100	99	100	99	100	102	104	103
Edge number	1060	1200	1185	1262	1037	1165	1239	1570
Positive edge	621	756	622	682	613	861	765	830
Negative edge	439	444	563	580	424	304	474	740
Average degree	21.200	24.240	23.700	25.490	22.734	21.182	22.734	29.074
Mean distance	1.831	1.840	1.832	1.809	1.853	1.888	1.853	1.789
Average clustering coefficient	0.974	0.975	0.929	0.976	0.970	0.975	0.970	0.976
Network density	0.211	0.242	0.248	0.253	0.210	0.194	0.210	0.272
Modularity	0.691	0.663	0.606	0.670	0.659	0.667	0.612	0.598

## Data Availability

The original data presented in the study are openly available in the NCBI Sequence Read Archive (SRA) under the accession number PRJNA1478890.

## Abbreviations

The following abbreviations are used in this manuscript:

SF	Shrub forest
DB	Deciduous broad-leaved forest
CB	Coniferous and broad-leaved mixed forest
CF	Coniferous forest
LMC	Litter moisture content
EC	Electrical conductivity
C	Carbon
N	Nitrogen
P	Phosphorus
Li	Lignin
Ce	Cellulose
He	Hemicellulose
ANOVA	One-Way Analysis of Variance
RDA	Redundancy Analysis
PCR	Polymerase Chain Reaction
DNA	Deoxyribonucleic Acid
